# Flexible, integrated, and person-centered psychiatric care through global treatment budgets: results of the multiperspective study PsychCare

**DOI:** 10.1007/s00115-025-01896-6

**Published:** 2025-09-18

**Authors:** Andrea Pfennig, Bettina Soltmann, Anne Neumann, Martin Heinze, Roman Kliemt, Dennis Häckl, Enno Swart, Fabian Baum, Yuri Ignatyev, Julian Schwarz, Denise Kubat, Ines Weinhold, Tarcyane Barata Garcia, Sebastian von Peter, Jochen Schmitt

**Affiliations:** 1 Department of Psychiatry and Psychotherapy, Faculty of Medicine and University Hospital Carl Gustav Carus, TUD Dresden University of Technology, Fetscherstraße 74, 01307 Dresden, Germany; 2 Center for Evidence-Based Healthcare, Faculty of Medicine and University Hospital Carl Gustav Carus, TUD Dresden University of Technology, Dresden, Germany; 3 Department of Psychiatry and Psychotherapy, Center for Mental Health, Immanuel Hospital Rüdersdorf, Brandenburg Medical School Theodor Fontane, Rüdersdorf, Germany; 4 Institute for Health Economics and Health Systems Research (WIG2) GmbH Leipzig, Leipzig, Germany; 5 Institute of Social Medicine and Health Systems Research, Otto-von-Guericke University Magdeburg, Magdeburg, Germany

**Keywords:** Mental health services, Psychiatric hospitals, Cross-sectoral treatment, Psychiatric care models, Satisfaction with care, Psychiatrische Dienste, Psychiatrische Krankenhäuser, Sektorenübergreifende Behandlung, Psychiatrische Versorgungsmodelle, Zufriedenheit mit der Versorgung

## Abstract

**Background:**

To overcome fragmented care provision in Germany, flexible, integrated psychiatric care (FIT) model projects according to § 64b of the German Social Code Book (SGB) V were implemented.

**Objectives:**

The results of the prospective cross-model, controlled, multiperspective/multimethod study PsychCare are presented and discussed along with data from statutory health insurance (SHI)-based research.

**Materials and methods:**

PsychCare applied a multi- and mixed-method design. Primary data were acquired in 18 psychiatric hospitals (*n* = 10 FIT; *n* = 8 matched treatment as usual—TAU) at study start (M-I) and 15 months later (M-II). Main outcomes were treatment satisfaction and health-related quality of life. Secondary outcomes included recovery, clinical decision-making, symptom severity, healthcare utilization and costs, needs and experiences with care, and caregiver burden. Participatory process evaluation assessed process-, structure-, and experience-related components.

**Results:**

Patients in FIT (*n* = 595) had significantly higher treatment satisfaction (ZUF-8: 26.3 ± 4.36 vs. 24.9 ± 4.70; *p* < 0.001) and recovery (RAS‑R total: 134 ± 35.8 vs. 119 ± 54.3; *p* < 0.001) at M‑I compared to TAU patients (*n* = 555), despite comparable symptom severity. About 50% of patients reported high satisfaction with clinical decision-making (*p* > 0.05); FIT caregivers were numerically more satisfied. Direct medical costs were significantly lower in FIT both at M‑I and M‑II. Type of care was associated with the degree of implementation of FIT components. Linking primary and SHI data was feasible.

**Conclusion:**

PsychCare showed that FIT was superior in cross-sectional treatment satisfaction, recovery, and caregiver satisfaction with suggested cost-effectiveness. Long-term FIT success compared to standard care needs further assessment.

**Supplementary Information:**

The online version of this article (10.1007/s00115-025-01896-6) includes further information and tables.

## Abstract (de)

**Hintergrund:**

Um die fragmentierte psychiatrische Gesundheitsversorgung in Deutschland zu überwinden, wurden flexible, integrierte Modellprojekte (FIT) auf der Grundlage des § 64b des Sozialgesetzbuchs (SGB) V implementiert.

**Ziele der Arbeit:**

Ergebnisse der prospektiven, modellübergreifenden, kontrollierten, multiperspektivischen/-methodischen Studie „PsychCare“ werden dargestellt und gemeinsam mit den Ergebnissen der Routinedatenauswertung diskutiert.

**Material und Methoden:**

„PsychCare“ nutzte ein multi- und mixed-methodisches Design. Primärdaten wurden in 18 psychiatrischen Kliniken (*n* = 10 FIT; *n* = 8 strukturell ähnliche Kliniken der Regelversorgung (TAU)) zum Studienstart (M-I) und 15 Monate später (M-II) erhoben. Primäre Zielgrößen waren die Behandlungszufriedenheit und die gesundheitsbezogene Lebensqualität. Sekundäre Zielgrößen umfassten Recovery, klinische Entscheidungsfindung, Symptomschwere, Inanspruchnahme von Versorgungsleistungen und Kosten, Beurteilung sektorenübergreifender Versorgung und Angehörigenbelastung. Die partizipative Prozessevaluation untersuchte Prozess‑, Struktur- und erlebensbezogene Versorgungsmerkmale.

**Ergebnisse:**

FIT-Patienten (*n* = 595) berichteten eine signifikant höhere Behandlungszufriedenheit (ZUF-8: 26,3 ± 4,36 vs. 24,9 ± 4,70; *p* < 0,001) und Recovery (RAS‑R total: 134,0 ± 35,8 vs. 119 ± 54,3; *p* < 0,001) zu M‑I verglichen mit TAU-Patienten (*n* = 555) bei vergleichbarer Symptomschwere. Knapp die Hälfte der Patienten berichtete von einer hohen Zufriedenheit mit der klinischen Entscheidungsfindung (*p* > 0.05), FIT-Angehörige waren numerisch zufriedener. Die direkten Kosten in FIT waren signifikant geringer. Da die gesellschaftlichen Kosten numerisch geringer und die primären Outcomes zu M‑I signifikant höher ausfielen, erschien FIT kosteneffektiv. Zu M‑II blieben die direkten Kosten in FIT signifikant geringer, keine weiteren Gruppenunterschiede wurden festgestellt. Der Versorgungstyp war assoziiert mit dem Umsetzungsgrad von prozess-, struktur- und erlebensbezogenen Versorgungsmerkmalen. FIT-Leistungserbringende benannten entökonomisierte Entscheidungsprozesse und hoben die Kooperation und gemeinsame Ziele mit den gesetzlichen Krankenkassen als zentrale Erfolgsfaktoren für die Umsetzung hervor. FIT verlangte dem Personal mehr Mobilität und Flexibilität ab. Die Verlinkung von primären und Kassendaten gelang.

**Diskussion:**

„PsychCare“ stellt die erste kontrollierte Evaluation von flexiblen, integrierten Modellprojekten auf der Grundlage des § 64b SGB V dar, welche umfangreiche Daten verschiedener Quellen einbezog. FIT war querschnittlich überlegen in Bezug auf die Behandlungszufriedenheit, Recovery sowie die Zufriedenheit der Angehörigen, Kosteneffektivität deutete sich an. Der langfristige Erfolg von FIT im Vergleich zur Regelversorgung sollte weiter untersucht werden.

## Background

Mental disorders constitute the main reasons for loss of healthy years of life worldwide and are associated with immense social and economic burden [8]. They often lead to permanent impairment in psychosocial functioning and health-related quality of life (HRQoL). The severity, chronicity, and frequent somatic and mental comorbidities are a challenge to the highly fragmented German healthcare system, underscoring the need to develop flexible, patient-oriented, and cross-setting care models [5].

With the introduction of the § 64b of the German social code book (SGB) V in 2012, psychiatric hospitals were enabled to provide cross-setting care based on a global treatment budget (GTB) negotiated with statutory health insurers (SHI). The aims of the resulting model projects are to establish a modern, cross-setting—at best cross-sectoral—(hospital-based) psychiatric care, to shift inpatient to outpatient and day-care settings, to improve the setting transfer, to efficiently use resources, to provide patient-centered care, and to involve the social milieu to a greater extent [18].

At the beginning of 2013, 25 model projects (FIT models = flexible and integrated treatment models) were launched in 11 German federal states. Within a specified range of the number of patients treated, FIT hospitals receive a fixed renumeration for treatment independent of the type, setting, or duration of care. Almost half of the FIT models were based on former contracts (i.e., models of integrated care according to § 140a SGB V or on GTB models for a region), others were developed de novo. Common FIT components include case managers, crisis resolution teams, home-based services, and cross-setting treatment groups. In four FIT hospitals, all SHI were part of the model contract; in the others, care was offered according to FIT and treatment as usual (TAU) in parallel depending on the SHI of the patient. Evaluations of the precursor contract models showed a reduced length of inpatient stay; however, studies often lacked independent evaluators, control groups, and/or consideration of cost-effectiveness [3, 6, 10, 11, 21, 22, 25, 27]. After initiating § 64b SGB V-based models, two cross-model evaluation studies were initiated: a controlled cohort study on the basis of SHI data (EVA64; [1, 13, 15]) and an uncontrolled patient- and provider-oriented exploratory study (EvaMod64b; [17, 18, 23]). However, a multiperspective, multimethod, and model-spanning evaluation approach integrating patient-reported outcomes (PRO) to compare FIT models with standard care was missing. PsychCare addresses this gap by assessing effects, costs, and cost-effectiveness from the perspectives of patients, caregivers, and providers. Additionally, a process evaluation was performed [19], experiences of the different interest groups regarding FIT were analyzed, and quality indicators were developed.

This article summarizes PsychCare results and discusses them in relation to SHI data to inform future healthcare planning.

## Methods

PsychCare was a controlled, prospective, multicenter cohort study conducted in 18 psychiatric hospitals across Germany (German Clinical Trial Register, DRKS 00022535). Study design details are described elsewhere [26]. Briefly, 10 of the 18 hospitals with § 64b SGB V contracts were selected by stratified random sampling (FIT). Stratification was based on contract-conclusion before (≥ 4 years of FIT experience) vs. in or after 2015 (newer models). Matched control hospitals (TAU) were identified using the algorithm applied in EVA64 [15, 20]. The 10 most suitable TAU sites per FIT were contacted consecutively. From February 2018 to September 2019, inpatient, day care, and outpatient patients (≥ 18 years of age) with a main diagnosis of schizophrenia, schizotypal disorder, delusional disorders or brief psychotic disorders, affective disorders or/and alcohol use disorders were screened consecutively for eligibility. These severe mental illnesses were selected as they often result in a recurrent or chronic course and constitute a significant proportion of hospital admissions.

**Fig. 1:**
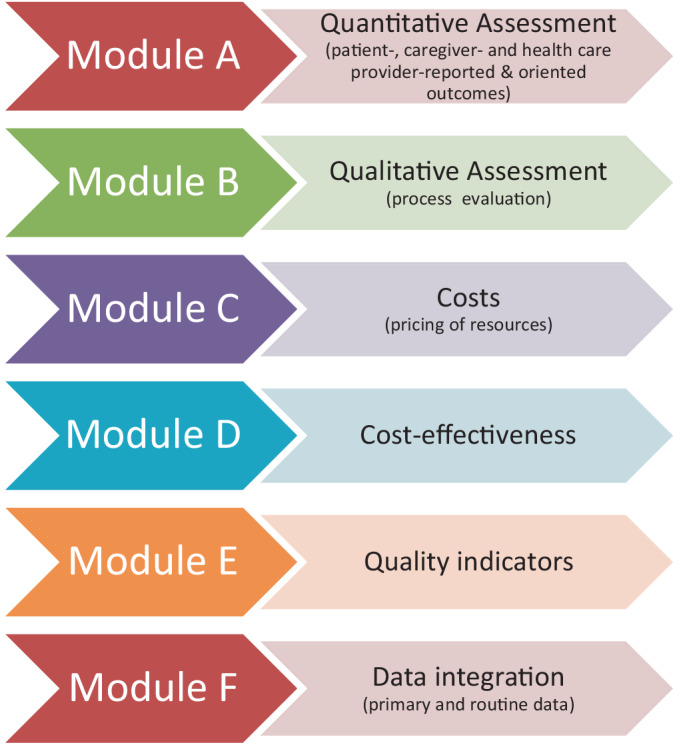
Study components of PsychCare

PsychCare consisted of six components, as shown in Fig. 1.

Readers are referred to Supplement S1 and [26] for a detailed description of the methods and instruments of the study components. *Quantitative primary data collection* was conducted at study start (M-I) and 15 months later (M-II; Module A). As FIT had already been implemented for at least 2 years in all model hospitals at study start, M‑I did not mark the start of model care. Therefore, it was decided prior to analysis to compare FIT and TAU at M‑I and M-II, rather than assess change over time. Treatment satisfaction and HRQoL were pre-defined primary outcomes; secondary outcomes included recovery, involvement, and satisfaction regarding clinical decision-making, symptom severity, healthcare utilization, assessment of cross-sectoral care, and caregiver burden (see Supplement S1 for instruments).

The *process evaluation* (Module B) used a mixed-methods approach. Based on two questionnaires assessing FIT-related components, researchers with and without personal experience of psychiatric treatment developed 12 experience-related components using a ground theory approach [9, 16]. From these, interview guidelines were developed and used in 71 problem-centered interviews. The standardized survey instrument Needs and Experiences in Psychiatric Treatment (NEPT) was developed and applied at M‑II to 374 patients [16]. Theory-guided guidelines for expert interviews and focus group assessments were developed, and 29 interviews were conducted with senior staff from the management and controlling departments and SHI in seven FIT. Overall, 400 h of participant observation were carried out at two FIT and one TAU hospital. The modules on *costs* (Module C) and *cost-effectiveness*** (**Module D) comprised a cost-effectiveness analysis (healthcare utilization, determination of costs, derivation of incremental cost-effectiveness ratios). An adapted version of the CSSRI (Client Sociodemographic and Service Receipt Inventory) was applied, and SHI data were used to validate self-report. The feasibility of individual *data linkage* of self-reported and SHI data was examined in Module F. Linkage was limited to patients who gave written informed consent to the usage of SHI data and, within this group, to those insured by one of the SHI participating in PsychCare (see [7]). As the development of *quality indicators* for a patient-centered, cross-setting mental health care (Module E) is still ongoing, results could not be presented in this article.

## Results

### Study population

**Fig. 2:**
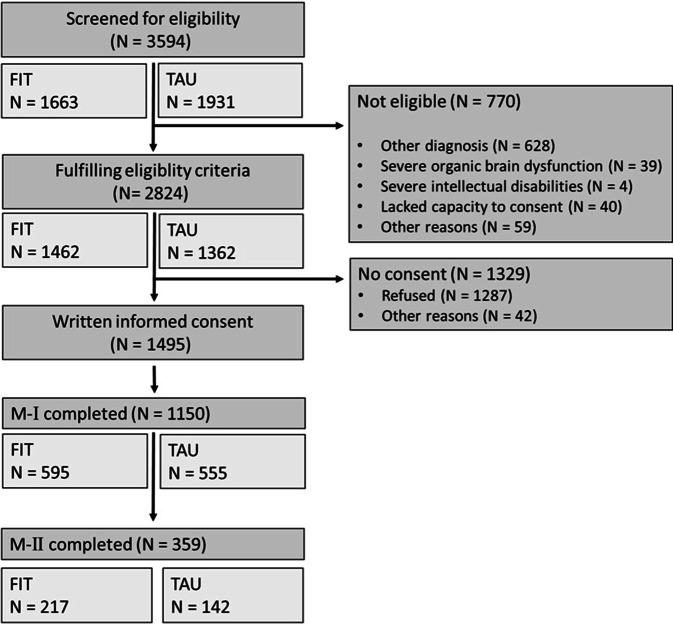
Flow chart of PsychCare. *FIT* flexible and integrated treatment, *TAU* treatment as usual

For the quantitative study, 3594 patients were screened, of whom 2824 (78.6%) fulfilled the inclusion criteria. From these, 1495 (52.9%) consented to participate (FIT: 51.2%, TAU: 54.6%). With a return rate of 81.0% in FIT and 76.9% in TAU, 1183 patients provided assessment data at M‑I (*n* = 1150 after reconciliation of discharge diagnoses and exclusion of incomplete questionnaires; FIT *n* = 595, TAU *n* = 555; see Fig. 2).

The sociodemographic and clinical characteristics of participants were comparable regarding age, sex, living circumstances, diagnoses, symptom severity, and duration of illness in FIT and TAU (see Supplement S2).

### Treatment satisfaction, recovery, decision-making, and caregiver burden

A significantly higher treatment satisfaction at M‑I (Patient Satisfaction Questionnaire [ZUF-8]: 26.30 ± 4.36 vs. 24.90 ± 4.70; *p* < 0.001) was found for FIT patients. The difference was statistically significant; however, it did not reach the study-specific pre-defined difference of two points assumed to indicate clinical relevance. At M‑II, while scores were numerically still higher in FIT, there was only a trend toward a difference (26.20 ± 4.34 vs. 25.40 ± 4.41, *p* = 0.095). At M‑I, HRQoL was also significantly higher in FIT patients (here reaching the pre-defined difference assumed to indicate clinical relevance), with again no statistical difference at M‑II [14].

At M‑I, patients in the FIT group showed a significantly higher recovery (RAS-R: 134.0 ± 35.8 vs. 119.0 ± 54.3; *p* < 0.001). This was also true for all five subscale scores. Again, at M‑II, there was no longer any difference (141.0 ± 36.9 vs. 141.0 ± 34.2, *p* = 0.864; Table 1).

**Table 1:** Recovery scores

		M‑I			M‑II		
		FIT (*N* = 595)	TAU (*N* = 555)	*p*	FIT (*N* = 217)	TAU (*N* = 142)	*p*
RAS‑R total score, mean (SD)		134 (35.8)	119 (54.3)	*<* *0.001*	141 (36.9)	141 (34.2)	0.862
Subscores, mean (SD)	Personal confidence and hope	27.1 (8.53)	24.3 (12.0)	*<* *0.001*	29.0 (8.73)	28.6 (8.66)	0.681
	Goal and success orientation	11.0 (3.54)	9.73 (4.74)	*<* *0.001*	11.3 (3.60)	11.5 (3.14)	0.512
	Willingness to ask for help	15.9 (5.19)	14.0 (7.09)	*<* *0.001*	16.4 (5.07)	16.6 (4.75)	0.745
	Reliance on others	13.6 (4.21)	12.2 (5.9)	*<* *0.001*	14.2 (4.28)	14.4 (3.84)	0.543
	No domination by symptoms	8.91 (3.46)	7.8 (4.32)	*<* *0.001*	10.0 (3.44)	9.64 (3.26)	0.283

*FIT* flexible, integrated treatment, *M‑I* measurement I, *M‑II* measurement II, *RAS‑R* Recovery Assessment Scale—revised, *SD* standard deviation, *TAU* treatment as usual

The reader is referred to Supplement S3 for the results of the regression models. Higher treatment satisfaction and recovery in FIT at M‑I were confirmed. Patients in day care and outpatients were more satisfied compared to inpatients. Outpatients showed higher recovery compared to inpatients. Patients with affective disorders seemed to be less satisfied with care and to have lower recovery scores compared to patients with a substance use disorder.

About half of the participants reported a high satisfaction with clinical decision-making (46.6% FIT vs. 50.3% TAU, M‑I), while only 6.0% and 8.2%, respectively, reported low satisfaction. The implementation of shared decision-making was high: 85.0% (FIT) and 80.2% (TAU) reported having made the decision together with their clinician or having taken actively part in the process at M‑I.

Caregivers of 113 FIT and 58 TAU patients were assessed at M‑I regarding their burden. In the three domains evaluated and at both time points, caregivers of FIT patients were numerically, but not statistically, more satisfied (see Supplement S4).

### Costs and cost-effectiveness

The direct medical costs at M‑I were significantly lower in FIT compared to TAU (15,667.1 €, SD = 17,475.1 € vs. 18,331 €, SD 17,722.4 €, *p* < 0.01). Patients in the FIT group were significantly less often in inpatient care (59.9% vs. 76.6%; *p* < 0.01), and the duration of inpatient care was significantly shorter (28.5 days, SD = 40.4 vs. 41.9 days, SD = 44.1; *p* < 0.01). There was no group difference in non-medical and indirect costs. Societal costs at M‑I were 21,200 € for FIT and 23,310 € for TAU (*p* = 0.058). At M‑II, societal costs were also lower in FIT than in TAU (19,336 € vs. 26,654 €; *p* = 0.093). The statistical significance level was not reached, potentially because of the sample size, particularly at M‑II, and the high variance, as reflected by the high standard deviations (sdFIT M‑I = 19,070 €; sdTAU M‑I = 19,991 €; sdFIT M‑II = 14,992 €; sdTAU M‑II = 17,159 €). With costs being numerically lower and ZUF‑8 and HRQoL scores significantly higher at M‑I, the results suggest the cost-effectiveness of FIT at M‑I. At M‑II direct medical costs were again significantly lower in the FIT group (5807.9 €, SD = 10,262.7 € vs. 9961.6 €, SD = 15,453.3 €; *p* = 0.005), again with a significantly shorter duration of inpatient treatment. No group differences were found with regard to societal costs, and cost-effectiveness could not be appraised due to the low number of individuals participating at that time point.

### Process evaluation

Good internal consistency was found for the NEPT (Cronbach’s α = 0.89). There was an increasing trend (*p* = 0.03) in the overall value across three independent groups based on the degree of FIT contract design, and the NEPT construct demonstrated a comparable validity to the ZUF‑8 construct. A significantly higher degree of implementation of the specific care components was observed for FIT compared to TAU (reported separately, [24]). They consistently exhibited higher averages reaching the significance level except for continuity of treatment team, multiprofessional collaboration, and sovereign steering of therapeutic measures (Tab. 2). In the mixed-method convergence design, clear associations emerged between hospital types and the degree of implementation of care components. In a two-dimensional representation, FIT with 100% FIT-contract design and/or long contract durations was distinctly separated from TAU and FIT that did not meet either criterion.

**Table 2:** Implementation of specific care components

Care components		FIT	TAU	Test	
No.		Mean (SD)	Mean (SD)	*p*	Cohens *d*
II	Flexibility in shifting settings	2.39 (1.01)	0.85 (0.87)	*0.010*	*1.62*
III	Continuity of treatment team	0.61 (0.35)	0.13 (0.13)	0.854	1.74
IV	Multiprofessional cooperation	2.10 (1.00)	1.54 (0.58)	0.223	0.67
V	Therapeutic group sessions across settings	2.00 (0.00)	1.00 (0.76)	*0.001*	*2.00*
VI	Outreach home-care	0.89 (0.33)	0.29 (0.49)	*0.017*	*1.48*
VII	Systematic involvement of informal caregivers	0.67 (0.49)	0.19 (0.18)	*0.011*	*1.24*
VIII	Accessibility of services	0.85 (0.17)	0.55 (0.18)	*0.004*	*1.76*
IX	Sovereign steering of therapeutic measures	0.68 (0.24)	0.37 (0.25)	0.087	1.23
X	Cooperation across sectors	0.71 (0.39)	0.29 (0.24)	*0.031*	*1.26*
XI	Expansion of professional expertise	0.63 (0.20)	0.14 (0.13)	*0.011*	*3.15*

*FIT* flexible, integrated treatment, *SD* standard deviation, *TAU* treatment as usual

Providers reported a de-economization of the clinical decision processes. The cooperation and shared aims of hospitals and SHI were seen to be central to the implementation. Participant observation revealed central challenges for realizing the transfer between the various service components: the spatial proximity/distance of the components, the realization of innovative alliances between these, and the information flow. Staff members perceived the mobility and flexibility needed as freedom and potential but also as an additional burden in terms of time.

### Data linkage

A large proportion of patients whose SHI was a partner in PsychCare agreed to the linkage of their self-reported and SHI data (89% FIT, 82% TAU). Relatively high absolute concordance on resource utilization was found across all settings, with higher values for inpatient and day care compared to outpatient services (see [7]).

## Discussion

PsychCare is the first controlled, prospective, multiperspective, and multimethod evaluation study for model projects based on § 64b of SGB V. Building on the two precursor studies—EVAMod64b” and “EVA64—it integrates the perspectives of patients, caregivers, and providers and combines primary with routine data.

### Main results of PsychCare

Compared to TAU, FIT was cross-sectionally superior with regard to treatment satisfaction and HRQoL [14], recovery, and caregiver satisfaction. The degree of implementation of specific care components for a flexible, patient-centered treatment was higher in FIT. Direct medical costs for FIT were significantly lower and FIT care seemed to be cost-efficient. Providers reported de-economization of the clinical decision processes. The cooperation and shared aims of hospitals and SHI were reported to be central to implementation. There was a higher demand for mobility and flexibility for the staff to realize the transfer between service components. Linkage of self-reported and SHI data was feasible, and a high proportion of patients agreed to this.

### Summarized results of the parallel SHI-based study EVA64

EVA64 found a significant reduction in the length of inpatient stay for patients initially treated in the hospital (no treatment in that hospital within the last 2 years) by about 5 days in FIT compared to TAU with a shift to day care and in some hospitals to outpatient treatment. The duration of sick leave, however, was not different in the groups [2, 12]. The odds for treatment continuity increased by 1.4 in FIT, indicating that the chance was higher for FIT patients to be seen in the psychiatric outpatient department and/or by a resident psychologist or psychiatrist within 30 days of discharge from hospital [13].

### Interpretation

The findings of superiority of FIT at M‑I regarding treatment satisfaction, HRQoL, recovery, and satisfaction of caregivers, as well as the implementation of flexible and integrated care components, confirmed the study hypotheses. The FIT and TAU hospitals were matched regarding regional as well as structural indicators, and the patient groups were comparable in major sociodemographic and clinical variables. At the outset of PsychCare, FIT hospitals had started FIT ≥ 2 years earlier and/or had applied a precursor FIT-like model treatment already (with one hospital having started a precursor model in 2003). Thus, M‑I differences in PsychCare are most likely attributable to ongoing model care performance. Assessing changes in outcomes over time is challenging, as care models are complex and component structures vary. Models of FIT function as learning systems, starting with an introductory phase and followed by process reorganization. Our study shows greater implementation of model components alongside the share of SHI funds included in the individual model contract. This suggests that key changes, i.e., in the attitude of staff (e.g., focusing more strongly on patient- and care-giver needs, working very close in cross-setting and cross-professional way, 24/7 availability) and structures (e.g., providing more day-care and flexible outpatient capacity, cross-setting group treatment) are more likely achieved when the majority of patients receive model care, rather than limiting it to those insured by individual SHI funds [23]. The government commission on a modern and needs-based hospital care in Germany [4] recommended that all SHI funds are to contract and evaluate model care programs that already exist in regions with ≥ 25% of the population. This would lead to a consistent regional regularization of psychiatric care and be an incentive to transfer the model to regional standard care.

Recovery and high HRQoL are key treatment aims: FIT patients showed better overall and component-specific recovery and reported higher HRQoL, supporting the prioritization of FIT-like care models in future mental health financing. Regression analyses showed that day-care patients and outpatients had higher satisfaction and recovery than inpatients. As FIT aims to increase flexibility based on patient needs, the shift toward day-care and outpatient treatment aligns with these findings.

The involvement of patients in treatment decision-making was high with at least 80% in both FIT and TAU groups, which is in line with guideline recommendations and shows that care outside model treatment provides components of flexible and integrated care as well; this is supported by the results of the comparison of component implementation during the process evaluation.

### Limitations

Group differences were mainly found only at M‑I, suggesting context variables that were difficult to capture. PsychCare coincided with the onset of the COVID-19 pandemic. The M‑I assessment was finished before the pandemic; however, 66% of M‑II assessments were conducted after the pandemic’s onset. Exploratory analyses suggested that FIT patients assessed after this time point reported a greater HRQoL decline relative to TAU than those assessed before the onset. Pandemic-related measures affected all aspects of healthcare, especially with day-care units often forced to close temporarily. As FIT hospitals shifted toward day and outpatient care, they had reduced inpatient capacity, potentially making them more vulnerable. Moreover, complex, interactive interventions may have been harder to adapt to crisis conditions or lost their distinctive character compared to routine care.

PsychCare was conducted mainly in non-academic hospitals, where research infrastructure is often limited. Limited resources because of burdening bureaucracy were reported to be a major reason for not taking part as a study center. Higher TAU rejection rates raise concerns about bias toward more motivated or progressive TAU sites, potentially inflating control group outcomes. Recruitment success varied by setting, with challenges especially in outpatient departments due to limited patient availability for additional study visits. Recruitment challenges are well-known in research within routine care and depend greatly on local structures, access, and the motivation of on-site recruiters. For the results of healthcare research to be representative, structural resources have to be supplemented in academic and non-academic settings. Participation in the M‑II measurement fell short of expectations, highlighting the challenge of conducting longitudinal studies in individuals with severe mental illness. With respect to the participation of caregivers, the two-stage inclusion process (patient recruitment, then caregiver recruitment) and the lack of close caregivers were reasons for the participation rate of only 19% and 10%, respectively.

Due to limited SHI participation, claims data linkage was only possible for a subset of participants, thus limiting the full potential of Module F. Nevertheless, high consent rates indicate that such linkage studies are feasible in psychiatric care.

Despite the aforementioned limitations and in line with the statements of our advisory board, we are confident that the data presented in this article should be considered in future healthcare planning.

## Practical conclusion

- Compared to treatment as usual (TAU), flexible and integrated psychiatric care (FIT) was superior regarding treatment satisfaction, recovery, and caregiver satisfaction with suggested cost-efficiency.
- Challenges included the assessment of change in ongoing projects, the impact of COVID-19, and recruitment in non-academic hospital settings.
- Despite FIT advantages seen at the first measurement point, its long-term success compared to TAU should be assessed further.
- In conjunction with the decreased duration of inpatient treatment, FIT hospitals seem to be superior in parameters important to patients, caregivers, providers, and statutory health insurance (SHI), and should be developed further to include all SHI and all outpatient treatment settings.

## Supplementary Information

Supplement S1: Methods of the study components

Supplement S2: Sociodemographic characteristics at M‑I

Supplement S3: Regression models on satisfaction with care and recovery

Supplement S4: Caregiver burden

## Data Availability

All data collected will be made available in anonymized form from the corresponding author on request.

## Declarations

The studies involving humans were reviewed and approved by the Institutional Review Board (IRB00001473 and IORG0001076) of the Faculty of Medicine of the TUD Dresden University of Technology and at each site where a separate approval was mandatory. The studies were conducted in accordance with the local legislation and institutional requirements. The patients/participants provided their written informed consent to participate in this study.
