# Supplementary material for: Flexible, integrated, and person-centered psychiatric care through global treatment budgets: results of the multiperspective study PsychCare
Source: Nervenarzt. 2025 Sep 18;96(6):542–50. doi: 10.1007/s00115-025-01896-6 (PMC12586395; doi:10.1007/s00115-025-01896-6)
Supplement: Supplementary file 1 — Supplement S1: Methods of the study components [file 115_2025_1896_MOESM1_ESM.pdf]

Supplement S1: Methods of the study components

A **quantitative primary data collection** was conducted at study-start (M-I) as well as 15 months later (M-II) (*Module A*). As FIT had been already been implemented in all model hospitals since at least two years at the time of patient recruitment, and hence M-I did not represent the start of model care, it was decided prior to the first analyses to compare FIT and TAU at M-I and M-II (and not change between time points as had been pre-defined). Health-related quality of life (HRQoL, QWB-SA, [15]) and treatment satisfaction (ZUF-8, German 8-item version of the Client Satisfaction Questionnaire [14]) were pre-defined primary outcomes, secondary outcomes included recovery (RAS-R, Recovery Assessment Scale - revised [3]), involvement in clinical decision-making (CDRC-P) and satisfaction with clinical decision-making (CDIS-P, [12, 16]), symptom severity (SCL-9, symptom severity checklist [17], healthcare utilization (D-CSSRI, Client Socio-Demographic and Service Receipt Inventory [13]), assessment of cross-sectoral care (NEPT - formerly SEPICC, [11]) and family burden (FBA; EUFAMI Part B, [1, 8]) (table S1). The hospital's study staff was trained in screening potential study participants, enrollment in the study and M-I assessment. M-II was conducted centrally via paper or phone assessment.

Sample size calculation for satisfaction with care resulted in n=110 patients each treatment and diagnosis subgroup (estimated effect size 0.39, difference 2.0, SD 5.1 – based on Bauer 2010, estimated loss-to-follow-up: 25%, α 5%; power: 80%). Appropriate two-sided tests for independent samples were used and unadjusted regression models calculated as cross-sectional comparison.

Table S1: Instruments of Module A (assessment at M-I and M-II)

	Outcome	Instrument	Theoretical scale range	Reference
**Primary**	Health related quality of life	QWB-SA	0-1	[15]
	Treatment satisfaction	ZUF-8	8 bis 32	[14]
**Secondary**	Symptom severity	SCL-9	9-45	[17]
	Recovery	RAS-R	41-205	[3]
	Healthcare utilization	CSSRI	n.a.	[13]
	Involvement in clinical decision-making and satisfaction with clinical decision-making	CDRC-P; CDIS-P	n.a.	[12, 16]
	Needs and Experiences in Psychiatric Treatment	NEPT (formerly SEPICC)	n.a.	[11]
	Family burden	FBA; EUFAMI Part B	n.a.	[1, 8]

QWB-SA: Quality of Well-being Scale self-administered scale; SCL-9: symptom checklist; SDQ: Strengths and Difficulties Questionnaire; RAS-R: recovery assessment scale; CSSRI: German Client Service Receipt Inventory; CDRC-P: Clinical Decision Making Style; CDIS-P: Clinical Decision Making Involvement and satisfaction; NEPT: Needs and Experiences in Psychiatric Treatment (=SEPICC); FBA: questionnaire on the burden of relatives; EUFAMI Part B: European Federation of Associations of Families of Mentally Ill People

The **process evaluation** (*Module B*) used a mixed-methods approach.

Based on two questionnaires assessing FIT-specific process and structure-related components (SEPICC and FITGrade), researchers with and without personal experience of mental crises and psychiatric treatment collaboratively developed 12 experience-related components using a Ground Theory approach [6, 9]. From these, interview guidelines were developed used in 71 problem-centered interviews with users from all included hospital settings. Analysis applied thematic analysis and a mixed method convergence design [10]. The experience-related components were further developed into a standardized survey instrument applied at M-II to n=374 patients (NEPT construct - Needs and Experiences in Psychiatric Treatment) [9].

To identify incentives, prerequisites and barriers for the implementation of FIT, theory-guided guidelines for expert interviews and focus groups assessments were developed and

29 interviews conducted with senior staff from the management and controlling departments and health insurance companies in seven FIT models. All interviews were recorded, anonymized, transcribed, and qualitatively content-analyzed [7].

A participant observation was carried out, focusing on the significance of various institutional and infrastructural regulations for the daily work of staff and how transfer between different settings of care was organized. In total, 400 hours of participant observations were carried out at three clinics (2 FIT, 1 TAU) by an ethnographer and analyzed using NVivo and a grounded theory methodology approach.

The modules on **costs** (*Module C*) **and cost-effectiveness (***Module D*) comprised a cost-effectiveness analysis, including assessment of health care utilization, determination of costs, and derivation of incremental cost-effectiveness ratios. Self-reported healthcare utilization was recorded with an adapted version of CSSRI [2, 13] up to 6 months prior to the interview. Statutory health insurance data (SHID) of participants who consented in using SHID were used to validate the self-reported data. For the cost-effectiveness analysis, the incremental cost-effectiveness ratio was calculated using quality adjusted life years (QALY) derived from QWB-SA values and costs of FIT and TAU [4, 5].

The feasibility of an individual **data linkage** of self-reported quantitative data and SHID was examined in the module on data integration (*Module F*). Linkage was limited to patients who gave written informed consent to the usage of SHID and, within this group, to those insured by one of the SHI participating in “PsychCare”.

The development of **quality indicators** (QIs) for a patient-centered, cross-setting mental health care (*Module E*) is still ongoing so that results could not be presented within this article.

References

1. Brand U (2001) European perspectives: a carer's view. Acta psychiatrica Scandinavica. Supplementum(410):96–101
2. Chisholm D, Knapp MR, Knudsen HC et al. (2000) Client Socio-Demographic and Service Receipt Inventory—European Version: development of an instrument for international research. EPSILON Study 5. European Psychiatric Services: Inputs Linked to Outcome Domains and Needs. The British journal of psychiatry. Supplement(39):s28-33
3. Corrigan PW, Salzer M, Ralph RO et al. (2004) Examining the factor structure of the recovery assessment scale. Schizophrenia bulletin 30(4):1035–1041
4. Drummond M, Sculpher M, Torrence G, O'Brien B, Stoddard G. (2005) Methods for the economic evaluation of health care programmes, 3. ed. Oxford medical publications. Oxford Univ. Pr, Oxford, New York, NY
5. Graf von der Schulenburg J-M, Greiner W, Jost F et al. (2007) Deutsche Empfehlungen zur gesundheitsökonomischen Evaluation - dritte und aktualisierte Fassung des Hannoveraner Konsens. Gesundh ökon Qual manag 12(5):285–290
6. Ignatyev Y, Timm J, Heinze M et al. (2017) Development and Preliminary Validation of the Scale for Evaluation of Psychiatric Integrative and Continuous Care-Patient's Version. Front. Psychiatry 8:162
7. Mayring P, Fenzl T (2019) Qualitative Inhaltsanalyse. In: Baur N, Blasius J (Hrsg) Handbuch Methoden der empirischen Sozialforschung, 2., vollständig überarbeitete und erweiterte Auflage. Springer VS, Wiesbaden, S 633–648
8. Möller-Leimkühler AM, Buchner E (2004) Belastungen und Bewältigungstile von Angehörigen schizophrener und depressiver Patienten. In: Möller H-J, Müller N (Hrsg) Schizophrenie: Langzeitverlauf und Langzeittherapie. Springer Vienna, Vienna, S 181–196
9. Peter S von (2017) Partizipative und kollaborative Forschungsansätze in der Psychiatrie (Participatory and Collaborative Strategies in Psychiatric Research). Psychiatrische Praxis 44(8):431–433
10. Peter S von, Jänchen P, Göppert L et al. (2023) Erlebensbezogene Qualitätsmerkmale für die psychiatrische Behandlung: Vorstellung eines vorläufigen multivariaten Konstrukts (Experience-based items of quality in psychiatric treatment: A first multivariate construct). Zeitschrift fur Evidenz, Fortbildung und Qualitat im Gesundheitswesen 176:51–60
11. Peter S von, Krispin H, Glück R et al. Needs and Experiences in Psychiatric Treatment (NEPT) – piloting a collaboratively gen-erated, initial research tool to evaluate cross-sectoral mental health services. in press
12. Puschner B, Becker T, Mayer B et al. (2016) Clinical decision making and outcome in the routine care of people with severe mental illness across Europe (CEDAR). Epidemiology and psychiatric sciences 25(1):69–79
13. Roick C, Kilian R, Matschinger H et al. (2001) Die deutsche Version des Client Sociodemographic and Service Receipt Inventory - Ein Instrument zur Erfassung psychiatrischer Versorgungskosten (German adaptation of the client sociodemographic and service receipt inventory - an instrument for the cost of mental health care). Psychiatrische Praxis 28 Suppl 2:S84-90
14. Schmidt J, Lamprecht F, Wittmann WW (1989) Zufriedenheit mit der stationären Versorgung. Entwicklung eines Fragebogens und erste Validitätsuntersuchungen. Psychother med Psychol 39:248–255.

15. Seiber WJ, Groessl EJ, David KM et al. (2008) Quality of well being self-administered (QWB-SA) scale, University of California, San Diego

16. Slade M, Jordan H, Clarke E et al. (2014) The development and evaluation of a five-language multi-perspective standardised measure. Clinical decision-making involvement and satisfaction (CDIS). BMC health services research 14:323

17. Wilkening A, Zeschky M, Ziegenbein M et al. (2007) Evaluation der Behandlungsergebnisse einer psychiatrischen Aufnahmestation. Ziele, Methodik und erste Ergebnisse eines Projektes zur Qualitätssicherung (Evaluation of therapy outcome on a psychiatric admission ward. Background, methods and first results of a project on quality management). Wiener klinische Wochenschrift 119(21-22):654–662
