# Supplementary material for: Flexible, integrated, and person-centered psychiatric care through global treatment budgets: results of the multiperspective study PsychCare
Source: Nervenarzt. 2025 Sep 18;96(6):542–50. doi: 10.1007/s00115-025-01896-6 (PMC12586395; doi:10.1007/s00115-025-01896-6)
Supplement: Supplementary file 2 — Supplement S2: Sociodemographic characteristics at M‑I [file 115_2025_1896_MOESM2_ESM.pdf]

**Supplement S2:** Sociodemographic characteristics at M-I (adapted from [27])

| | FIT (N=595) | TAU (N=555) |
|---|---|---|
| **Age** | | |
| Mean ± SD | 45.7 ± 14.2 | 47.5 ± 15.2 |
| **Age groups (y)** | | |
| 18-39 | 208 (35.0%) | 182 (32.8%) |
| 40-59 | 303 (50.9%) | 269 (48.5%) |
| ≥ 60 | 82 (13.8%) | 104 (18.7%) |
| **Sex** | | |
| Female | 312 (52.4%) | 271 (48.8%) |
| **Partnership status** | | |
| Married / co-habiting | 142 (23.9%) | 132 (23.8%) |
| **Accommodation** | | |
| Supported accommodation | 22 (3.7%) | 21 (3.8%) |
| **Living situation** | | |
| Living alone | 257 (43.2%) | 242 (43.6%) |
| **CASMIN**[1] | | |
| University degree | 91 (15.3%) | 88 (15.9%) |
| Secondary school leaving certificate/abitur and vocational education | 96 (16.1%) | 73 (13.2%) |
| Secondary school leaving certificate/abitur without vocational education | 26 (4.4%) | 27 (4.9%) |
| Lower secondary school leaving certificate and vocational education | 163 (27.4%) | 155 (27.9%) |
| Lower secondary school leaving certificate without vocational education | 28 (4.7%) | 20 (3.6%) |
| Main school leaving certificate and vocational education | 83 (13.9%) | 70 (12.6%) |
| Main school leaving certificate without vocational education | 44 (7.4%) | 53 (9.5%) |
| Currently in training | 39 (6.6%) | 37 (6.7%) |
| No completed training | 12 (2.0%) | 14 (2.5%) |
| **Diagnosis (ICD-10)** | | |
| F 10 | 125 (20.6%) | 138 (24.5%) |
| F 20-23 | 117 (19.2%) | 85 (15.1%) |
| F3 | 353 (58.1%) | 332 (59.0%) |

[1]nach Brauns H, Scherer S, Steinmann S (2003) The CASMIN Educational Classification in International Comparative Research. In: Hoffmeyer-Zlotnik JHP, Wolf C (Hrsg) Advances in Cross-National Comparison. Springer US, Boston, MA, S 221–244) (ex-post from acquired data)

| | FIT (N=595) | TAU (N=555) |
|---|---|---|
| **Treatment duration** | | |
| ≤ 5 y | 207 (34.8%) | 196 (35.3%) |
| > 5 y | 388 (65.2%) | 359 (64.7%) |
| **Setting at M-I** | | |
| Inpatient | 170 (28.6%) | 404 (72.8%) |
| Day care | 292 (49.1%) | 121 (21.8%) |
| Outpatient | 129 (21.7%) | 12 (2.2%) |
