# Supplementary material for: Flexible, integrated, and person-centered psychiatric care through global treatment budgets: results of the multiperspective study PsychCare
Source: Nervenarzt. 2025 Sep 18;96(6):542–50. doi: 10.1007/s00115-025-01896-6 (PMC12586395; doi:10.1007/s00115-025-01896-6)
Supplement: Supplementary file 3 — Supplement S3: Regression models on satisfaction with care and recovery [file 115_2025_1896_MOESM3_ESM.pdf]

Supplement S3: Regression models on satisfaction with care and recovery

	Satisfaction with care				Recovery			
	M-I		M-II		M-I		M-II	
	estimate	p-value	estimate	p-value	estimate	p-value	estimate	p-value
**Group** (ref: TAU)								
FIT unadjusted	1.44	**<0.001**	0.83	0.095	15.00	**<0.001**	0.66	0.864
**Age groups** *(ref: 18-39 y)*								
40 – 59 y	1.09	**<0.001**	-0.24	0.683	3.54	0.232	5.85	0.191
≥ 60 y	0.84	**0.047**	0.43	0.600	-1.10	0.786	1.91	0.763
**Sex** *(ref.: male)*								
female	0.34	0.228	-0.,41	0.426	1.43	0.591	-2.90	0.460
**Diagnosis** (ICD-10) *(ref: F10)*								
F20-23	-0.05	0.906	-0.56	0.504	-8.42	0.051	2.28	0.729
F30-39	-0.27	0.431	-0.69	0.315	-11.95	**<0.001**	-4.44	0.391
**Setting at M-I** (ref: inpatient)								
Day care	1.11	**<0.001**			2.38	0.459		
outpatient	1.31	**0.007**			10.96	**0.018**		
**Chronic co-morbidity**[1] *(ref: none)*								
≥ 1	-0.57	0.079	0.58	0.317	-1.45	0.641	-7.67	0.089
**Treatment duration** *(ref: ≤ 5 y)*								
> 5 y	-0.37	0.191	0.31	0.579	-0.76	**0.006**	-10.34	**0.015**

Ref: ICD-10: International Classification of Diseases, 10th revision, FIT: flexible, integrated treatment, M-I: measurement I, M-II: measurement II, ref: reference, TAU: treatment as usual, y: years

[1]Nach Domenech C, Pastore A, Altamura AC, Bernasconi C, Corral R, Elkis H, et al. Correlation of Health-Related Quality of Life in Clinically Stable Outpatients with Schizophrenia. Neuropsychiatr Dis Treat. 2019;15:3475-86.; Huber MB, Felix J, Vogelmann M, Leidl R. Health-Related Quality of Life of the General German Population in 2015: Results from the EQ-5D-5L. Int J Environ Res Public Health. 2017;14(4).
