# Supplementary material for: Flexible, integrated, and person-centered psychiatric care through global treatment budgets: results of the multiperspective study PsychCare
Source: Nervenarzt. 2025 Sep 18;96(6):542–50. doi: 10.1007/s00115-025-01896-6 (PMC12586395; doi:10.1007/s00115-025-01896-6)
Supplement: Supplementary file 4 — Supplement S4: Caregiver burden [file 115_2025_1896_MOESM4_ESM.pdf]

Supplement S4: Carer's burden

		M-I		M-II	
		**FIT (N=113)**	**TAU (N=58)**	**FIT (N=36)**	**TAU (N=12)**
Satisfaction with…					
mean (SD)	Information and tips	3.05 (0.88)	2.93 (0.89)	3.66 (0.843)	3.60 (2.03)
	Involvement in treatment and care / care planning	3.10 (1.36)	2.85 (1.08)	3.41 (1.01)	3.13 (1.07)
	Support from doctors / nurses	3.16 (0.78)	2.98 (0.84)	3.62 (1.10)	3.19 (0.929)

FIT: flexible, integrated treatment, M-I: measurement I, M-II: measurement II, SD: standard deviation, TAU: treatment as usual
